# Case report on novel mutation in SPAST gene in Polish family with spastic paraplegia

**DOI:** 10.1186/s12883-019-1561-6

**Published:** 2019-12-14

**Authors:** Aleksandra Klimkowicz-Mrowiec, Anna Dziubek, Malgorzata Sado, Marek Karpiński, Agnieszka Gorzkowska

**Affiliations:** 1 Department of Neurology, Jagiellonian University, School of Medicine, 31-503 Krakow, Botaniczna 3, Krakow, Poland; 2 Department of Neurology, University Hospital, Krakow, Poland; 3 Department of Neurology, Department of Neurorehabilitation, Medical University of Silesia, Katowice, Poland

**Keywords:** Hereditary spastic paraplegia, SPAST, Novel mutation, Clinical phenotype

## Abstract

**Background:**

Hereditary spastic paraplegia is a large group of degenerative, neurological disorders characterized by progressive lower limb spasticity and weakness. The disease was investigated precisely but still clinicians often make incorrect or late diagnosis. Our aim was to investigate the genetic background and clinical phenotype of spastic paraplegia in large Polish family.

**Case presentation:**

A 37 years old woman presented with 4-year history of walking difficulties. On neurological examination, she had signs of upper motor lesion in lower extremities. She denied sphincter dysfunction and her cognition was normal. Her family history was positive for individuals with gait problems. The initial diagnosis was familial spastic paraplegia. Genetic testing identified a novel mutation in *SPAST* gene. All available family members were examined and had genetic testing. The same mutation in *SPAST* gene was identified in other affected family members. All patients caring the mutation presented with different phenotypes.

**Conclusion:**

This study presents a family with spastic paraplegia due to a novel mutation c.1390G›T(p.Glu464Term) in *SPAST* gene. Affected individuals showed a range of phenotypes that varied in their severity. This case report demonstrates, the signs of hereditary spastic paraplegia can be often misdiagnosed with other diseases. Therefore genetic testing should always be considered in patients with lower limb spasticity and positive family history in order to help to establish the correct diagnosis.

## Background

Hereditary spastic paraplegias (HSPs) are a rare and heterogeneous group of neurodegenerative disorders characterized by slowly progressive spasticity and weakness of lower extremities. Its prevalence varies from 1.2 to 9.6/100000 [1].

HSPs are classified by the phenotype, the trait of inheritance and the mutated gene. Based on the phenotype, HSPs are classified as pure when spastic paraplegia is the only symptom or complex when it is associated with other clinical features like involvement of upper extremities, cognitive impairment and behavioral changes.

The most commonly involved are *SPAST* and *ATL1* genes. Mutations in the *SPAST* on chromosome 2p22.3 account for 15–40% of all autosomal dominant HSPs cases [2]. It encodes the protein spastin, a member of the ATPase associated with diverse cellular activity family with a role in microtubule dynamics.

Clinically, HSPs due to mutations in *SPAST,* present as a pure form in most cases but phenotypic variations are also reported [3].

Strategy for exploration of gene of interest is guided by clinical presentation, model of inheritance, and age of onset. In our case, the pure clinical form, autosomal dominant pattern of inheritance, and age of onset above 20 years led us to begin with exploring *SPAST* gene. Genetic studies identified a new mutation in AAA-domain of *SPAST* gene. To confirm the pathogenicity of this mutation we used computational predictive programs and additionally we assessed co-segregation in affected family members. Those who had signs of upper motor lesion and had been previously diagnosed with other diseases, carried the same mutation.

## Case presentation

**Fig. 1:**
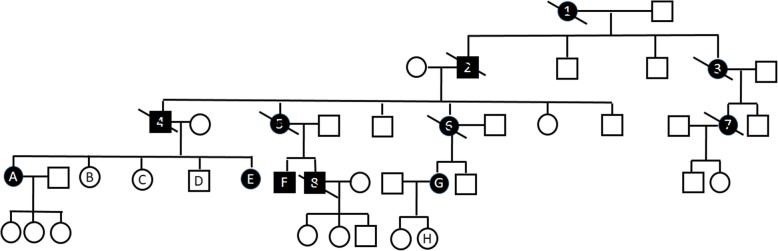
Pedigree of a family with spastic paraplegia. Black circles (females) and squares (males) represent affected people with spastic paraplegia. Cross-hatched symbols represents affected individuals already deceased (1,2,3,4,5,6,7,8). Unaffected individuals are not shaded. Individuals A,B,C, D, E, F, G, H had neurological and genetic evaluations

A 37 years old woman (E) presented with walking difficulties. The first symptoms began 4 years prior to presentation when she noticed walking more slowly. She had no other complaints, denied previous and current use of drugs. Some other members of her family also experienced gait problems. (Fig. 1 presents the pedigree of the family. Table 1. presents characteristics of family members.)

**Table 1:** Characteristic of study participants

Patient	A	E	F	G	1	2	3	4	5	6	7	8
Age at examination (years)	56	37	57	51	n/a	n/a	n/a	n/a	n/a	n/a	n/a	n/a
Age at onset of symptoms	n/a	33	37	39	›30	~ 42	›30	~ 45	›40	›43	›30	~ 42
Age at death	n/a	n/a	n/a	n/a	›50	72	›65	66	79	72	›65	47
Motor symptoms	non	Mild gait disorder, slow deterioration	Very severe gait disorder, slow deterioration	Severe gait disorder, slow deterioration	Severe gat disorder, slow deterioration	Severe gait disorder, slow deterioration	Severe gait disorders	Severe gait disorders	Mild gait disorder, slow deterioration	Severe gait disorder, slow deterioration	Severe gait disorders	Mild gate disorders, frequent falls
Neurological examination	Bilateral hyperreflexia, Babinski sign	Spastic para-paresis	Spastic para-paresis	Spastic para-paresis	n/a	n/a	n/a	n/a	n/a	n/a	n/a	n/a
Spastic Paraplegia Rating Scale	0	14	29	23	n/a	n/a	n/a	n/a	n/a	n/a	n/a	n/a
Disability stage	No mobility problems	Mild gait stiffness	Moderate gait stiffness Uses crane age 55	Moderate gait stiffness Occasionally needs support when walking	n/a	Moderate gait stiffness Used bilateral support above age 60 when walking	n/a	In wheel-chair 6 years before death	Moderate gait stiffness Needed bilateral support when walking 10 years before death	Used crane age 67	n/a	n/a
Medical conditions complicating disability			Polio disease in childhood						Stroke 3 years before death	›43 after her mother death she developed depression, stayed at home, was not active. She broke leg at age 60 and since then she lead sedentary lifestyle and then was bed-ridden for few years		Abuse of alcohol/liver cancer

*n/a* Not available

On neurological examination, she had spastic gait, symmetrical proximal weakness of the lower extremities (quadriceps and gluteal muscle), mild spasticity in hamstrings, quadriceps, adductors, gastrocnemius, and soleus. Increased deep tendon reflexes and Babinski sign were present bilaterally. She denied sphincter dysfunction. Her cognition was normal.

Laboratory tests did not reveal any abnormalities in blood count, electrolytes value, coagulation test, renal, hepatic or thyroid functions. C-reactive protein was ‹ 1 mg/L. Vitamin B12 level was normal. The nerve conduction study and evoked visual potentials were normal. Spinal and brain MR showed normal appearance of corpus callosum and did not find any cause of pyramidal syndrome. She was diagnosed with progressive pyramidal syndrome exclusively involving lower extremities. Her initial diagnosis was HSP.

After a written consent was obtained from the patient, the blood was taken for genetic testing. Molecular DNA analysis of the *SPAST* gene was performed. The Sanger sequencing method revealed mutation in one allele of *SPAST* gene: c.1390G›T(p.Glu464Term). This variant was not registered in the following genetic public database: HGMD, LOVD, NCBI ClinVar, dbSNP and ExAC. To determine pathogenicity in silico of this variant we used online prediction programs: Mutation Taster (http://www.mutationtaster.org/), SIFT/Provean (http://provean.jcvi.org/index.php), PolyPhen-2 (http://genetics.bwh.harvard.edu/pph2/index.shtml). Also, the blood samples for DNA testing were collected from 7 other available family members. The work was conducted in accordance with the Declaration of Helsinki.

The same mutation was identified in 3 other probands (A,F,G). The pathogenicity of the novel mutation found in this study was categorized according to the guidelines of the American College of Medical Genetics and Genomics [4]. Under this criteria this mutation was classified as pathogenic (Criteria: PVS1, PM1, PM2, PM4).

Patient (A), 56 years old woman, did not complain of any neurological symptoms or gait difficulties but presented with brisk reflexes and bilateral Babinski sign on neurological examination.

Patient F, 57 years old woman, suffered from polio disease when he was 8, with right hemiparesis and full recovery after a few months. At the age of 37, he experienced increasing walking difficulties. He was diagnosed with post-polio syndrome. At the age of 39 he was unable to work as a carpenter.

On neurological examination, he had spastic gait with heel strike progressively shifted forward, and asymmetrical proximal weakness of the lower extremities, more advanced on the right one. Severe spasticity in quadriceps, hamstrings, thigh adductor muscles, gastrocnemius and soleus was also found. Increased deep tendon reflexes in lower limbs and Babinski sign were present bilaterally. He denied sphincter dysfunction. His cognition was normal.

Another family member (G), 51 years old man, started to experience problems in her late thirties. She was admitted to the hospital in 2007. Cerebrospinal fluid analysis and laboratory tests did not reveal any abnormalities. The nerve conduction study was normal. The cervical and thoracic MR shoved disc herniation with suspicion of spinal cord suppression on multiple levels. Patient was discharged from the hospital with diagnosis of lower limb spasticity due to disc herniation: Th6/Th7 and Th8/TH9 and qualified for neurosurgery. Before agreeing to the surgery she sought neurosurgery consultation in another clinic. She had another thoracic spine MR, confirming multilevel disc herniation, but the signal of the spine was not changed, therefore she was disqualified from operation. Since that time, she observed slow progression of disability despite constant rehabilitation.

On neurological examination, she had spastic gait, symmetrical weakness of the lower limbs, mild spasticity in hamstrings, quadriceps, adductors, gastrocnemius, and soleus. Increased deep tendon reflexes in lower limbs and Babinski sign were present bilaterally. She did not report any sphincter dysfunction. Her cognition was normal.

The other genotyped family members (B,C,D,H) had no clinical and neurological symptoms and signs, and did not carry the mutation.

Our observational study indicates that novel variant c.1390G›T(p.Glu464Term) in *SPAST* gene is associated with HSP.

Patient has provided informed consent for publication of the case.

## Discussion and conclusions

The novel mutation was identified in the AAA-domain of *SPAST, region where most identified so far variants were located and still new studies confirm this observation* [5]*.*

As described before, the clinical expression of mutations in *SPAST* gene within a family may vary from asymptomatic patients, mildly affected individuals to severely affected patients [3]. Our study confirms this observation; the phenotype of affected family members varied from absence of clinical symptoms and complains to severe disability. The onset of symptoms was above 30 years of age in all cases, there was no association with sex and affected parent age at onset, what is consistent with previous studies [6].

To summarize, our study presents a family with a novel mutation in *SPAST* gene. The affected family members presented with different clinical phenotypes. The medical history of affected subjects shoves that clinical symptoms characteristic for HSP can be misdiagnosed with other diseases. Therefor in patients with spastic paraparesis and positive family history for gait problems, the HSP should be suspected at the top of differential diagnosis list and genetic testing should be considered.

## Data Availability

All data generated or analyzed during this study are included in this published article.

## Abbreviations

HSP: Hereditary spastic paraplegia
MR: Magnetic resonance imaging
